# Geometric Analysis of a System with Chemical Interactions

**DOI:** 10.3390/e23111548

**Published:** 2021-11-21

**Authors:** Dmitry Gromov, Alexander Toikka

**Affiliations:** 1 Faculty of Applied Mathematics and Control Processes, St. Petersburg State University, 199034 St. Petersburg, Russia; 2 Department of Chemical Thermodynamics and Kinetics, Institute of Chemistry, St. Petersburg State University, 199034 St. Petersburg, Russia; a.toikka@spbu.ru

**Keywords:** chemical reactions, contact geometry, isoaffine manifolds, stability conditions

## Abstract

In this paper, we present some initial results aimed at defining a framework for the analysis of thermodynamic systems with additional restrictions imposed on the intensive parameters. Specifically, for the case of chemical reactions, we considered the states of constant affinity that form isoffine submanifolds of the thermodynamic phase space. Wer discuss the problem of extending the previously obtained stability conditions to the considered class of systems.

## 1. Introduction

The study of thermodynamic stability is one of the main and traditional tasks of general and chemical thermodynamics. The general stability criteria were proposed by Gibbs [1] at the end of 19th century, but further investigations showed the usefulness and significance of other various forms of thermodynamic stability conditions, especially for the systems subject to complex external influences and specific restrictions.

With reference to Gibbs, the deviations from equilibrium are typically assumed to be virtual, although in his fundamental work [1], Gibbs discussed the possibility of real deviations or, specifically, fluctuations. In Prigogine’s later work [2], the analysis of stability conditions was carried out taking into account fluctuations, but the inclusion of such elements really requires a significant expansion of the theory, as was noted by Tisza [3]. The analysis of fluctuations should include finite deviations, and this opens up the possibility to consider the difference between stable and metastable states. On the other hand, such a consideration requires knowledge of the complete state diagram of the system state, and general conclusions cannot be obtained, cf. Münster [4]. Already Gibbs noted that an extension of his stability conditions to finite perturbations would require defining the boundaries within which they will be valid: “…it must be possible to assign limits within which it shall hold true of finite differences” [1], (p. 106).

Gibbs also proposed a variant of the stability condition that transforms the stability matrix into the product of second derivatives of internal energy w.r.t. the extensive variables at different fixings of the sets of intensive and extensive parameters [1], (p. 114). A thorough analysis of these conditions was performed in the papers [5,6] based upon a contact geometric formulation. The geometric framework has proved to be rather helpful for analyzing complex thermodynamic relations. Specifically, several additional results have been obtained within this framework—in particular, those related to the Le Chatelier–Brown principle. It was also indicated how these conditions can be used to conclude about the stability of a thermodynamic system.

In this paper, we aim at extending the developed approach to the thermodynamic systems with additional restrictions imposed on the intensive parameters. The most important, and by far the most practically relevant problem statement is presented by chemical reactions. In this case, the condition of constant affinity defines the isoaffine submanifolds of the thermodynamic phase manifold, thus resulting in a natural geometric formulation of the problem.

It is worth noting that the idea of using a geometric approach to analyzing thermodynamic relations was also suggested by Gibbs at the end of 19th century. Later, Hermann [7] recognized a deep connection between the equilibrium energy manifold and a Legendre manifold of the specific 1-form, later referred to as the thermodynamic 1-form. This contact geometry-based approach had been recognized and further developed in a number of papers [8,9,10]; see also more recent papers [11,12,13,14,15] and references therein. Almost concurrently with the first papers on contact geometric description of thermodynamic systems, the first substantial work on applying the geometric approach to chemical reaction dynamics was carried out by Oster, Perelson and Katchalsky in the 1970’s; see, e.g., Oster and Perelson [16]. Later, van der Schaft and Maschke [17] extended these results and formulated the dynamics of reaction networks within the port-Hamiltonian framework.

A somewhat different approach consists in studying the structure of chemical reactions as determined by the stoichiometric matrix. This matrix possesses a number of nontrivial properties and has been subject to a thorough study for decades; see [18,19] for an overview of recent results. Quite remarkably, within the framework developed in [5,6], the matrix theoretic analysis of chemical reactions turns out to be well connected to the geometric study of the isoaffine submanifolds. This connection will be demonstrated below.

### Notation

Throughout the paper, we use upright capital letters to denote matrices, slanted capital vectors for thermodynamic variables and functions, and small letters for auxiliary parameters and constants. Vectors are denoted either by upright capital letters, e.g., Y or X, or by lower-case bold letters, e.g., x, depending on the context. We write 0 and 1 to denote the vector (matrix) all of whose elements are equal to 0, resp. 1. It is assumed that the dimensions of all matrices are clear from the context. Otherwise, the dimensions are written as lower subscripts, e.g., $0_{\left[2\times k\right]}$.

Let U(X), $U:R^{n}\to R$, be a sufficiently smooth function. We let ∇U and $\nabla^{2}U$ denote the row vector of partial derivatives of U(X) (the gradient), and the [n×n] matrix of second-order partial derivatives of U(X) (the Hessian). Let $M\in R^{\left(n\times m\right)}$ be a rectangular matrix; we then define the null and the range spaces as $N\left(M\right)=\left\{X\in R^{m}|MX=0\right\}$ and $R\left(M\right)=\left\{Y\in R^{n}|Y=MX,X\in R^{m}\right\}$, respectively. Respectively, the co-range and the co-null spaces are $coR\left(M\right)=N\left(M^{⊤}\right)$, and $coN\left(M\right)=R\left(M^{⊤}\right)$. Note that for a symmetric matrix, the range and co-null spaces coincide, as well as the null and co-range spaces. Furthermore, we write M[I,J] to denote a submatrix of the [n×n] matrix M, obtained by removing from M the rows with indices i∈I⊂{1,…,n} and the columns with indices j∈J⊂{1,…,n}. The notation M[I,∅] (resp., M[∅,J]) means that only rows (resp., columns) are removed.

## 2. Results

### 2.1. Geometric Representation of the Energy Manifold

Let U(Y) be a sufficiently smooth first-order positive homogeneous function of *n* variables $Y\in R_{+}^{n}$. We define *n* conjugate variables X as $X_{i}=\frac{\partial U(Y)}{\partial Y_{i}}$.

Within the thermodynamic context, the function U(Y) is referred to as the internal energy function, the vector $Y={\left[S,\;V,\;N_{1}\;\ldots ,\;N_{n-2}\right]}^{⊤}$ contains the extensive parameters *S*, *V*, $N_{i}$ (entropy, volume, and the molar numbers of substances, respectively), while the vector $X={\left[T,\;-P,\;\mu_{1}\;\ldots ,\;\mu_{n-2}\right]}^{⊤}$ contains *n* intensive parameters *T*, −P, $\mu_{i}$ (temperature, pressure, and chemical potentials of respective components). In the following, we will mostly use the generic notations $Y_{i}$ and $X_{i}$ for the extensive and intensive variables, respectively, and will use the specific notation *S*, *T* etc. to emphasize the role of particular variables. We also note that our notation implies that there are n−2 different components (substances); hence we assume that n≥4 to ensure that the problem is not degenerate.

The evolution of the system can be represented by the trajectory of a point on a co-dimension 1 manifold in the space of n+1 variables $\left(Y_{0},\;Y\right)$, where $Y_{0}=U\left(Y\right)$. The position of this point is uniquely specified by the values of n+1 extensive variables $\left(Y_{0},\;Y\right)$ (note that $Y_{0}=U$ is an extensive variable as well because it is determined by a first-order homogeneous function). However, for the purpose of formal analysis, it is desirable to have a formulation that explicitly encompasses not only the extensive, but also the intensive variables X.

In [5,6], the authors proposed a systematic approach to the analysis of thermodynamic systems based upon the apparatus of contact geometry. Below, we will present a brief overview thereof.

The suggested approach consists in embedding the *n*-dimensional energy manifold into a (2n+1)-dimensional space with coordinates $\left(Y_{0},\;Y,\;X\right)$, which is referred to as the thermodynamic phase space. The energy equilibrium manifold U (which can be formally seen as a Legendre submanifold of a particular thermodynamic 1-form, see Gromov and Caines [20] for details) is thus defined by the following system of equations:

(1) $$\left\{\begin{array}{c} \phi_{0}=Y_{0}-U\left(Y_{1},\ldots ,Y_{n}\right)=0\\ \phi_{1}=X_{1}-\frac{\partial U}{\partial Y_{1}}\left(Y_{1},\ldots ,Y_{n}\right)=0\\ \vdots \\ \phi_{n}=X_{n}-\frac{\partial U}{\partial Y_{n}}\left(Y_{1},\ldots ,Y_{n}\right)=0. \end{array}\right.$$

System (1) can be compactly written as $\Phi (Y_{0},Y,X)=0$, where $\Phi (Y_{0},Y,X)$ is a smooth vector-valued function, $\Phi :R^{2n+1}\to R^{n+1}$. The representation (1) along with the implicit function theorem can be used to derive many useful thermodynamic relations, as was demonstrated in [5].

The application of the implicit function theorem is based upon the computation of the Jacobian matrix of $\Phi (Y_{0},Y,X)$. We write it concisely as

(2) $$D\Phi \left(Y_{0},Y,X\right)=\left[\begin{array}{cccc} 1\; & | & -\nabla U\;| & \;0\\ 0\; & | & -\nabla^{2}U| & \;I \end{array}\right],$$

where the symbols ∇ and $\nabla^{2}$ are used to denote the gradient (the row-vector of partial derivatives) and the Hessian (the matrix of second-order partial derivatives) of the energy function *U*, respectively. The matrix DΦ has a full row rank, i.e., rankDΦ=n+1. Note that the matrix $\nabla^{2}U$ has a rank equal to n−1 as follows from the Gibbs–Duhem equation (see [5] for the formal derivation).

Let the point $\Xi =({\bar{Y}}_{0},\;\bar{Y},\;\bar{X})$ belong to the equilibrium energy manifold U, i.e., it holds that Φ(Ξ)=0. Thus, according to the implicit function theorem, in a neighborhood of Ξ one can express n+1 variables as functions of the remaining *n* variables. However, the application of the implicit function theorem goes far beyond that. It allows one to compute the partial derivatives of some dependent parameters with respect to certain independent parameters for different choices of independent parameters, thus providing a detailed characterization of the energy manifold curvature. In [6], such calculations were used to derive equilibrium conditions for a thermodynamic system not undergoing a chemical reaction.

### 2.2. Geometry of an Isoaffine Submanifold

We wish to expand the developed framework by considering the setup, where there are chemical interactions between the components of the system. Suppose that the individual components are involved in *k* reactions, k<n−2. Each reaction is described by the balance equation that for the *i*th reaction reads as follows:

$$\sum_{j=1}^{n-2}\nu_{ij}R_{j}=0,$$

where $R_{j}$ are the chemical reagents (species), and $\nu_{ij}$ are the stoichiometric coefficients, which are used to balance the reaction (positive values for reaction products and negative for initial reactants). The entropy production σ for a unit volume *V* due to this non-equilibrium process is presented as follows [2]:

$$\sigma =\sum_{i=1}^{k}\frac{A_{i}}{T}\frac{1}{V}\left(\frac{d\xi_{i}}{dt}\right),$$

where $\xi_{i}$ is the extent of the *i*th reaction, *t* is time, and $A_{i}$ is the affinity of the *i*th reaction, which is defined as:

(3) $$A_{i}=\sum_{j=1}^{n-2}\nu_{ij}\mu_{j}.$$

Affinity $A_{i}$ is associated with the thermodynamic force, while the velocity of the *i*th reaction, $\frac{d\xi_{i}}{dt}$, is interpreted as the flow. Accordingly, the progression of the *i*th reaction can be characterized both by $\xi_{i}$ and $A_{i}$. However, the extent of the *i*th reaction is not a thermodynamic variable [4]: it describes only the deviation from the equilibrium. In contrast to the extent of reaction, chemical affinity, defined by (3), is a thermodynamic variable (a function of chemical potentials). Formally, affinity of a reaction can be determined at any stage of the reaction by introducing an inhibitor (negative catalyst), that is, by “freezing” the reaction [4]. This implies that one can consider the states of constant affinity, i.e., isoaffine submanifolds of the thermodynamic phase space, which are defined by the equations $A_{i}=const.$

We are specifically interested in studying the properties of the system when the chemical reactions are in equilibrium, i.e., when affinity is zero. Alternatively, this can be formulated as: $\sum_{j=1}^{n-2}\nu_{ij}\mu_{j}=0$, which can be concisely written in vector-matrix notation as

(4) Σμ=0.

Here, $\mu ={\left[\mu_{1},\ldots ,\mu_{n-2}\right]}^{⊤}$ is the vector of chemical potentials, and $\Sigma =[\nu_{ij}]$, i=1,…,k, j=1,…,n−2 is the matrix of stoichiometric coefficients. We assume that there are k<n−2 independent reactions, which implies that the matrix Σ has full row rank, i.e., rankΣ=k. Note that the assumption of zero affinity is not restrictive in any way, as all of the subsequent analysis will be focused on the structure of the stoichiometric matrix, which remains unchanged.

The conditions (4) determine an isoaffine submanifold $U_{A}$ of the energy manifold U, which we will later refer to as the *isoaffine energy manifold*. To analyze the dimensionality of this manifold one has to check the rank of the Jacobian matrix of the extended system of constitutive equations formed by (1) and (4). We denote the extended system by $\tilde{\Phi }$ and write the Jacobian as:

(5) $$D\tilde{\Phi }\left(Y_{0},Y,X\right)=\left[\begin{array}{ccccc} 1\; & | & -\nabla U\; & | & \;0\\ 0\; & | & -\nabla^{2}U & | & \;I\\ 0\; & | & 0 & | & \;\tilde{\Sigma } \end{array}\right],$$

where $\tilde{\Sigma }=\left[0_{\left[k\times 2\right]},\;\Sigma \right]$, i.e., we pad Σ with two zero columns. Obviously, both Σ and $\tilde{\Sigma }$ have the same row rank.

The co-dimension of the manifold $U_{A}$ at a point $\Xi =(Y_{0},Y,X)$ is equal to the rank of $D\tilde{\Phi }\left(Y_{0},Y,X\right)$ at that point. One can easily see that the rank of $D\tilde{\Phi }$ is determined by the rank of the principal submatrix obtained by removing the first row and the column of $D\tilde{\Phi }$. The following theorem provides the required characterization.

**Theorem** **1.** *Consider the [(n+k)×2n] matrix*

(6) $$M=\left[\begin{array}{cc} -\nabla^{2}U & I\\ 0 & \tilde{\Sigma } \end{array}\right],$$

*where $\tilde{\Sigma }\in R^{\left(k\times n\right)}$ is a full row rank matrix, i.e., $\mathrm{rank}\tilde{\Sigma }=k<n-2$, and $\nabla^{2}U$ is a symmetric matrix with a rank deficiency equal to 1, i.e., $\mathrm{rank}\nabla^{2}U=n-1$. Then, the rank of M is equal to n+k−1 if $N\left(\tilde{\Sigma }\right)\subset R\left(\nabla^{2}U\right)$ and n+k otherwise.*

**Proof.**  See Appendix A. □

To interpret the formulated theorem we recall that the null space $N(\nabla^{2}U)$ changes as the system state moves along the manifold and coincides with $\alpha \bar{Y}$, α≠0, at a point $\Xi =(Y_{0},\bar{Y},\bar{X})$. Furthermore, note that $R\left(\nabla^{2}U\right)⊥N\left(\nabla^{2}U\right)=\bar{Y}$ as $\nabla^{2}U$ is a symmetric matrix. Thus the condition $N\left(\tilde{\Sigma }\right)\subset R\left(\nabla^{2}U\right)$ is equivalent to saying that $N\left(\tilde{\Sigma }\right)⊥\bar{Y}$, which, in turn, implies that ${\bar{Y}}^{⊤}$ belongs to the span of the rows of matrix $\tilde{\Sigma }$. Note, however, that the first two elements of each row of the matrix $\tilde{\Sigma }$ are zero, which implies that the manifold $U_{A}$ loses its rank at the points where ${\bar{Y}}_{1}={\bar{Y}}_{2}=0$, which do not belong to the energy manifold U. Thus, we eventually conclude that the manifold $U_{A}$ has a (full row) constant rank equal to n+k+1, where one dimension comes from the removed first row.

## 3. Dependent and Independent Thermodynamic Variables on an Isoaffine Energy Manifold

The analysis carried out in the previous subsection implies that on the manifold $U_{A}$ one can choose n+k+1 dependent variables that can be expressed through the remaining n−k ones. To put it differently, in a small neighborhood of any point $\Xi \in U_{A}$, one can choose exactly n−k independent coordinates.

To provide an intuition for this result, assume that we have chosen variables (S,V,μ) as independent ones and the remaining ones as dependent. If the variables μ are consistent with (4), one can express *k* chemical potentials in terms of the remaining (n−2−k) ones. Thus, the set of independent variables reduces to n−k: *S*, *V* and (n−k−2) independent chemical potentials. However, the problem of determining which variables can serve as independent ones is in general rather intricate as will be discussed below.

In the following, we will assume that the internal energy *U* is considered as a dependent variable. We thus restrict ourselves to considering 2n thermodynamic variables Y and X. The problem of determining which variables can be taken as dependent boils down to finding n+k linearly independent columns of the [2n×2n] matrix (6). The first *n* columns correspond to the extensive variables Y, while the remaining columns correspond to the intensive variables X.

One can immediately observe that—in contrast to the case without chemical reactions—the intensive variables have a different status: while the variables *T* and −P are “free”, the chemical potentials $\mu_{j}$ are “bound” by (4). On the other hand, the chemical potentials are not in equal rights as well: some of them can be expressed through others, while some cannot. One should also take into account that a chemical potential, in our case the internal energy *U*, is determined up to an arbitrary constant; this is the property of any potential.

Note that the value of $\mu_{j}$ is usually defined through the standard value for the pure component *j* at certain constant values of *T* and *P*:

$$\mu_{j}=\mu_{j}^{\circ }\left(T,P\right)+\mu_{j}^{mix},$$

where $\mu_{j}^{mix}$ is the change in the chemical potential as a result of mixing the original pure substance with other substances at given *T* and *P*. Thus, the affinity (3) is also a function of *T* and *P*. Formally, an additional link between chemical potentials, temperature and pressure could also be imposed by assuming that, e.g.,

$$A_{i}=T+P-\sum_{j=1}^{n-2}\nu_{ij}\mu_{j}=const.,$$

but such constructions go beyond the scope of realistic physical framework. The change in the standard value $\mu_{j}^{\circ }\left(T,P\right)$ can also be derived from the Gibbs–Helmholtz equation

$$G=H+T{\left(\frac{\partial G}{\partial T}\right)}_{P}\;,$$

where *G* is the Gibbs energy and *H* is enthalpy. For pure substance, the molar Gibbs energy is equal to the chemical potential, but the determination of this value requires knowledge of the temperature dependence of enthalpy. As a result, no general conclusions can be obtained along this line.

Before proceeding with the detailed analysis, observe that the whole set of extensive variables Y cannot be chosen to be dependent, as the columns of the matrix $\nabla^{2}U$ are linearly dependent. However, under the thermodynamic stability condition, cf. [5], any [(n−1)×(n−1)] principal submatrix of $\nabla^{2}U$ has full rank. This allows us to formulate the first result.

**Theorem** **2.** *Let J⊂{1,n−2} be the set of indices of k linearly independent columns of the matrix Σ. Then, the set of n+k dependent variables can be chosen as the union of one of the sets $\left\{T,V,N_{1},\ldots \right\}$, $\left\{S,-P,N_{1},\ldots \right\}$ or $\left\{T,-P,N_{1},\ldots \right\}$, and the set of chemical potentials ${\left\{\mu_{j}\right\}}_{j\in J}$.*

**Proof.**  See Appendix A. □

Note that the set of linearly independent columns of the matrix Σ is not uniquely determined. Thus, depending upon the structure of the matrix Σ, the set *J* can be chosen in different ways, which introduces additional degrees of freedom.

Theorem 2 can be extended as the following result shows.

**Theorem** **3.** *Let J⊂{1,n−2} be the set of indices of k linearly independent columns of the matrix* Σ. *Then, the set of n+k dependent variables can be chosen as the union of the set of chemical potentials ${\left\{\mu_{j}\right\}}_{j\in J}$ and the set $Y=\{S,V,N_{1},\ldots \}$ with some elements from the set {S,V} replaced by the respective elements from {T,−P}, and up to n−2−k variables $N_{i}$, i∈{1,…,n−2}∖J replaced by the respective variables $\mu_{i}$.*

**Proof.**  See Appendix A. □

Note that in all preceding theorems, the set of chemical potentials ${\left\{\mu_{j}\right\}}_{j\in J}$ was always chosen to be dependent. These variables can be chosen to be independent as well, but this choice would not be valid for the whole isoaffine submanifold. A characterization of the subsets of $U_{A}$, where the respective columns of the Hessian matrix (6) turn out to be linearly dependent, is closely related to the problem of stability analysis of the respective system and will be reported in detail in a subsequent publication.

## 4. Discussion

In this paper, we presented some initial results aimed at defining a framework for the analysis of systems with additional restrictions imposed on the intensive parameters. Specifically, for the case of chemical reactions, we considered the equilibrium states or, alternatively, the states of constant affinity as the most important setup for physical chemistry. However, using the developed approach, one can consider the situations where some other links, e.g., between *T* and *P*, are imposed.

The presented results form a rigorous mathematical framework for the formulation and analysis of stability conditions in the course of chemical reactions, including chemical equilibrium. We emphasize that we are not discussing the stability of the chemical equilibrium itself, but rather the stability of the system as a whole with additional coupling equations (in this case, for the constancy of chemical affinity). These and similar problems have been considered in some works in the traditional thermodynamic way and for specific cases. Some examples may be the works of Gitterman for chemical equilibrium [21,22], Prigogine on configurational heat capacity (e.g., [2,23]), or the recent work by Toikka [24]. However, it is believed that the presented formulation would allow for a more systematic analysis of the problems of stability and equilibrium displacement using well-developed methods of vector algebra and matrix analysis.

## Data Availability

All necessary data are contained in the paper.

## Appendix A. Proofs of the Theorems

**Proof of Theorem 1.** Note that $\mathrm{rank}M=2n-\dim\left(N(M)\right)$. Therefore, it suffices to compute the dimension of the null space of M. Consider the vectors $x=\left[\begin{array}{c} x_{1}\\ x_{2} \end{array}\right]\in R^{2n}$, where $x_{1},x_{2}\in R^{n}$. A vector x belongs to N(M) if Mx=0, which amounts to the following system:

(A1a) $$\begin{array}{cc} -\nabla^{2}Ux_{1}+x_{2}= & \;0 \end{array}$$

(A1b) $$\begin{array}{cc} \;\;\;\;\Sigma x_{2}= & \;0. \end{array}$$

By definition, there are exactly (n−k) linearly independent vectors $x_{2}$ satisfying (A1b), $\{x_{2}^{i}\},\;i=1,\ldots ,n-k$. For each such vector, the system (A1a) has a solution if $x_{2}\in R\left(\nabla^{2}U\right)$. If this is the case, we have $N\left(\Sigma \right)\subset R\left(\nabla^{2}U\right)$, that is, the null space of Σ forms a linear subspace of the range space of $\nabla^{2}U$. Note that k<n−2 and hence, this is always possible. Let us pick up a set of (n−k) orthogonal vectors $x_{1}^{i}$ satisfying (A1a) for the corresponding $x_{2}^{i}$. The set of vectors $x^{i}$, obtained by vertically stacking the vectors $x_{1}^{i}$ and $x_{2}^{i}$, forms an (n−k)-dimensional subspace of $R^{2n}$. This set can be extended by the vector $x^{⊥}=\left[\begin{array}{c} x_{1}^{⊥}\\ 0 \end{array}\right]$, where $x_{1}^{⊥}\in N\left(\nabla^{2}U\right)$. This vector is linearly independent with the previously defined vectors as $x^{⊥}⊥coR\left(\nabla^{2}U\right)∋x_{1}^{i}$. Noting that $\dim(N\left(\nabla^{2}U\right))=1$, we conclude that there are no more linearly independent vectors that can be added to the =set. Therefore, dim(N(M))=k+1 and rank(M)=2n−(n−k+1)=n+k−1. This proves the first part of the statement. To prove the second part, we note that since $\mathrm{rank}\nabla^{2}U=n-1$, $N\left(\Sigma \right)⊄R\left(\nabla^{2}U\right)$ implies $N\left(\Sigma \right)+R\left(\nabla^{2}U\right)=R^{n}$. Using the Grassmann theorem, we conclude that dim(N(Σ)$\cap R(\nabla^{2}U))=1$. The latter implies that there are (n−1) linearly independent vectors $x_{2}^{i}\in N\left(\Sigma \right)$ satisfying (A1a) for some (linearly independent) vectors $x_{1}^{i}\in R\left(\nabla^{2}U\right)$. Extending this set by the vector $x^{⊥}$, we obtain a set of (n−k) linearly independent vectors that form the basis of N(M). Computing the dimensions, we obtain the required result. □

**Proof of Theorem 2.** Denote by S the matrix, formed by *j*th columns of Σ, j∈J, i.e., S=Σ[∅,N∖J], where N={1,…,n}. Furthermore, suppose that we replace $Y_{1}=S$ by $X_{1}=T$. The remaining two cases are shown in the same way. We need to show that the matrix

$$M=\left[\begin{array}{ccccc} 1\; & | & -\nabla^{2}U\left[\emptyset ,1\right]\; & | & 0\\ 0\; & | & \; & | & I\\ 0\; & | & 0 & | & \;S \end{array}\right],$$

is full rank. The result follows immediately by noting that M is a block triangular matrix and $\mathrm{rank}\nabla^{2}U\left[1,1\right]=\mathrm{rank}\nabla^{2}U\left[\emptyset ,1\right]$ (see ([5] Thm. A1), also [25]). □

**Proof of Theorem 3.** To prove the stated theorem, assume that the extensive variables $\left(Y_{3},\ldots ,Y_{n}\right)=\left(N_{1},\ldots ,N_{n-2}\right)$, and the respective intensive variables $\left(X_{3},\ldots ,X_{n}\right)=\left(\mu_{1},\ldots ,\mu_{n-2}\right)$ are renamed in the way that the last *k* variables correspond to the linearly independent columns of the matrix Σ. Now let us replace the first q<n−k elements of Y by the respective elements $\left(X_{1},\ldots ,X_{q}\right)$. Denote Q={1,…,q}. Then, the variables $\left(X_{1},\ldots ,X_{q},Y_{q+1},\ldots ,Y_{n}\right)\cap \left(X_{k},\ldots ,X_{n}\right)$ form a set of dependent variables if the following matrix is non-singular:

$$P=\left[\begin{array}{ccccc} I_{k}\; & | & -\nabla^{2}U\left[\emptyset ,Q\right]\; & | & \;0_{\left[(n-k)\times k\right]}\\ 0_{\left[(n-k)\times q\right]}\; & | & \; & | & I_{k}\\ \tilde{S}\; & | & 0_{\left[k\times (n-q)\right]} & | & \;S \end{array}\right],$$

where $\tilde{S}$ is the [k×q] matrix, formed by the first q<n−k columns of $\tilde{\Sigma }$ that correspond to the variables being replaced, and S is the matrix formed by the last *k* (linearly independent) columns of $\tilde{\Sigma }$. One can show that the leading *n*-principal submatrix of P in non-singular using the same arguments as in the proof of Theorem 2, and S is non-singular by definition. The matrix P is non-singular if the Schur complement of the matrix S, denoted as P/S, is non-singular (see [26] Section 0.8.5). Thus, we need to check the following matrix:

$$P/S=[\begin{array}{cc} I_{k}\; & |\\ 0_{\left[(n-k)\times q\right]}\; & | \end{array}-\nabla^{2}U\left[\emptyset ,Q\right]\;]-\left[\begin{array}{c} 0_{\left[(n-k)\times k\right]}\\ I_{k} \end{array}\right]S^{-1}\left[\begin{array}{ccc} \tilde{S}\; & | & \;0_{\left[k\times (n-q)\right]} \end{array}\right].$$

Note that q<n−k and hence,

$$\left[\begin{array}{c} 0_{\left[(n-k)\times k\right]}\\ I_{k} \end{array}\right]S^{-1}\left[\begin{array}{ccc} \tilde{S}\; & | & \;0_{\left[k\times (n-q)\right]} \end{array}\right]=\left[\begin{array}{c} 0_{\left[(n-k)\times k\right]}\\ I_{k} \end{array}\right]\left[\begin{array}{ccc} S^{-1}\tilde{S}\; & | & \;0_{\left[k\times (n-q)\right]} \end{array}\right]=0.$$

We conclude that the Schur complement P/S is non-singular, and the matrix P is non-singular as well. □
